# Short-term storage of tiger salamander (*Ambystoma tigrinum*) spermatozoa: The effect of collection type, temperature and time

**DOI:** 10.1371/journal.pone.0245047

**Published:** 2021-01-11

**Authors:** Amanda B. Gillis, Emmet L. Guy, Andrew J. Kouba, Peter J. Allen, Ruth M. Marcec-Greaves, Carrie K. Kouba

**Affiliations:** 1 Department of Biochemistry, Molecular Biology, Entomology, and Plant Pathology, Mississippi State University, Mississippi State, MS, United States of America; 2 Department of Wildlife, Fisheries, and Aquaculture, Mississippi State University, Mississippi State, MS, United States of America; 3 National Amphibian Conservation Center, Detroit Zoological Society, Royal Oak, MI, United States of America; University Hospital of Münster, GERMANY

## Abstract

The aims of this project were to characterize tiger salamander (*Ambystoma tigrinum*) spermatozoa motility over time, when excreted as either milt or spermic urine prior to packaging into a spermatophore, and to determine the effect of temperature on sperm motility. A split-plot design was utilized to assess the motility of the two pre-spermatophore sample types at two temperatures, 0°C and 20°C (n = 10 for each treatment). Spermiation was induced through exogenous hormone treatment of luteinizing hormone releasing hormone analog in order to collect both milt and spermic urine, which were evaluated for motility, divided into two separate aliquots, and subsequently stored in either an ice-bath (0°C) or on the benchtop (20°C). The decay rate of sperm motility was assessed by reevaluating subsamples at 0.5, 1, 2, 3, 5, 7, and 24 hours following the initial assessment. Results showed that sperm stored at 0°C had significantly higher progressive, non-progressive, and total motility for both sperm collection types over time. An interaction was found between collection type and time, with milt exhibiting lower initial motility that was more sustainable over time, compared to spermic urine. For both milt and spermic urine, motility decreased rapidly with storage duration, indicating samples should be used as soon as possible to maximize motility for *in-vitro* fertilization and cryopreservation. This is the first study to describe the differences in sperm motility between milt and spermic urine from an internally fertilizing caudate and demonstrates the benefits of near freezing temperatures on sperm longevity.

## Introduction

Currently, 41% of amphibians are threatened due to habitat loss, overexploitation, pollution, and emerging diseases [1]. While captive breeding, release, and relocation programs have been put in place over the last several decades, only 9% of threatened species are represented in captive assurance colonies worldwide [1, 2]. Additionally, only 14 reintroduction programs are considered highly successful following release of captive bred individuals [2]. This low success rate is primarily because captive breeding for many amphibians can be challenging due to an absence of environmental cues to stimulate reproduction, mate incompatibility, or asynchronous production of gametes leading to poor fertilization [3]. Assisted reproductive technologies (ARTs), such as hormone therapy, *in-vitro* fertilization (IVF) and gamete cryopreservation, can help overcome these barriers by circumventing the missing environmental cues necessary for reproduction. In the case of frogs and toads (order: Anura), ARTs have been beneficial for numerous captive breeding and reintroduction programs of threatened species, including the boreal toad *Anaxyrus boreas*, Mississippi gopher frog *Lithobates sevosus*, northern Corroboree frog *Pseudophryne pengilleyi*, Puerto Rican crested toad *Peltophryne lemur*, and Wyoming toad *Anaxyrus baxteri* [4, 5]. Unfortunately, for salamanders (order: Caudata) ARTs are lagging and have not been effectively incorporated into any breeding programs, which is particularly alarming as more than 52% of caudates are threatened with extinction [1].

**Fig 1:**
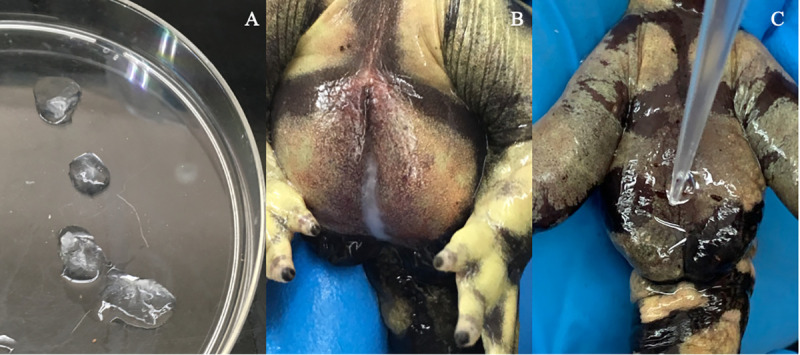
Collection types. Forms of semen that can be collected from internally fertilizing caudates following hormone induction include (A) spermatophore, (B) milt, and (C) spermic urine.

There are several potential reasons behind the lack of ART development for caudates including geographic distribution, fertilization strategy, and low representation in captive collections. For example, the majority of caudate biodiversity is located in North America (endemism > 30%); thus, other parts of the world like Australia, where ARTs are being developed for anurans, have no native salamanders or newts. In addition, their poor sustainability and low visibility as display animals have discouraged many zoos from establishing captive breeding programs. Perhaps the greatest hurdle is that the majority of salamanders and newts (excluding family Hynobiidae, Cryptobranchidae, and presumably Sirenidae) all display internal fertilization, whereas external fertilization is observed in most anurans [6]. In anurans, IVF is straightforward and mimics the natural behavior of a male directly depositing spermic urine on externally laid eggs [3]. While in caudates, the male naturally deposits a spermatophore (Fig 1A), which is a gelatinous packet of spermatozoa. The female collects and stores the spermatophore in a cloacal structure called a spermatheca, until the eggs are fertilized as they pass through the cloaca during oviposition [6].

This reproductive strategy is difficult to replicate with ART because of the invasiveness of artificial insemination and the need to mechanically or chemically break down the spermatophore for IVF. However, sperm can also be artificially collected in other forms such as milt and spermic urine [7, 8]. Milt is described to be a milky, viscous sample with a high concentration of spermatozoa, while spermic urine is a clear, free-flowing solution comprised of mostly urine and lower concentration of sperm (Fig 1B and 1C) [7]. Both sample types can be collected following hormone administration and prior to final spermatophore packing, with milt more frequently obtainable right after treatment (0.5 hour), while spermic urine is more commonly collected at later time points [7]. In general, sperm expressed as milt and spermic urine are easier to manipulate than when embedded in the gelatinous matrix of the spermatophore capsule. Analysis of sperm motility and concentration, or use of the sperm for IVF, requires dissolution of the capsule to free the sperm from the immobilizing matrix, whereas the fluid nature of the milt and spermic urine is already suited to these applications. Moreover, the spermatophore’s gel matrix limits cryoprotectant permeability compared to milt or spermic urine for long term cryostorage of gametes.

However, a distinction has rarely been made between the two pre-spermatophore sample types, milt and spermic urine, when exogenous hormone-stimulation was utilized to induce sperm expression [9–12], with the exception of studies by Marcec [8]. Furthermore, milt and spermic urine have not been differentiated in their unique physiological or biochemical characteristics beyond the descriptions above.

Once milt or spermic urine is collected for IVF or cryopreservation, an understanding of the duration of gamete viability at different temperatures and over time is necessary to establish optimized protocols for assisted breeding and gamete storage. Anuran sperm viability and motility is typically short-lived once expressed into a low osmotic environment (e.g. urine or water) at ambient temperatures, prompting development of methods to extend motility and viability, such as cold storage (0–4°C), aeration or addition of antibiotics [3, 5, 13–16]. Unlike anurans, the time frame for which caudate sperm remains motile has been examined in only a few studies with a wide range of outcomes within families including Ambystomatidae [8, 9], Cryptobranchidae [10, 12, 17] and Salamandridae [11, 18]. In Ambystomatidae, the effect of storage temperature on sperm motility has yet to be formally investigated, especially when examined based on collection type, to determine the working timeframe for ART application. Therefore, the purpose of this study was to: 1) evaluate the relationship of sperm motility over time at two different storage temperatures; and 2) determine if sample type (milt or spermic urine) impacts the time and temperature decay rates for motility. The tiger salamander (*Ambystoma tigrinum*) is the most widespread salamander in North America and is currently listed as least concern by the IUCN Red List [19, 20]. Thus, tiger salamanders serve as an excellent model for other Ambystomid mole salamanders (e.g. *A*. *californiense*, *cingulatum* and *bishopi*), which are exclusively endemic to North America and are the second most species-rich caudate family in the United States [20]. In addition, due to their size and internal fertilization, they are exceptional models to develop ARTs before application to other threatened species.

## Materials and methods

### Experimental animals

Sexually mature, terrestrial, male tiger salamanders (n = 27; Mean ± SE, age (yr) = 2.9 ± 1.0; total length (cm) = 20.8 ± 0.6; weight (g) = 42.6 ± 2.6) were held at Mississippi State University (33.453880 latitude, -88.794090 longitude). Animals were either purchased from private breeders or produced in the laboratory through IVF. Salamanders were housed in tanks (30 cm x 46 cm x 66 cm) in groups of 3–6 animals on a substrate mixture of organic soil and coconut fiber approximately 3 cm deep. A water dish, ceramic plate, and PVC tubing refugia were furnished for each tank. In addition to ambient room lighting, type B ultraviolet light was provided for each tank and set on a timer to mimic the light cycle within the tiger salamander’s natural range within Mississippi. Ambient temperature averaged 20.5°C year-round. Animals were fed an alternating diet of crickets (*Gryllodes sigillatus*), mealworms, and Dubia roaches (*Blaptica dubia*) three times a week and were supplemented with calcium (Zoo Med Laboratories, Inc., CA, USA) and a vitamin mix (Supervite, Repashy Ventures Inc., CA, USA) once a week by lightly dusting the insects immediately prior to feeding [21]. Prior to hormone treatment, food was withheld for two to three days to prevent regurgitation and fecal contamination of samples [8]. All husbandry and experimental protocols were reviewed and approved by the IACUC at Mississippi State University (#17–189).

### Exogenous hormone treatment and experimental design

**Fig 2:**
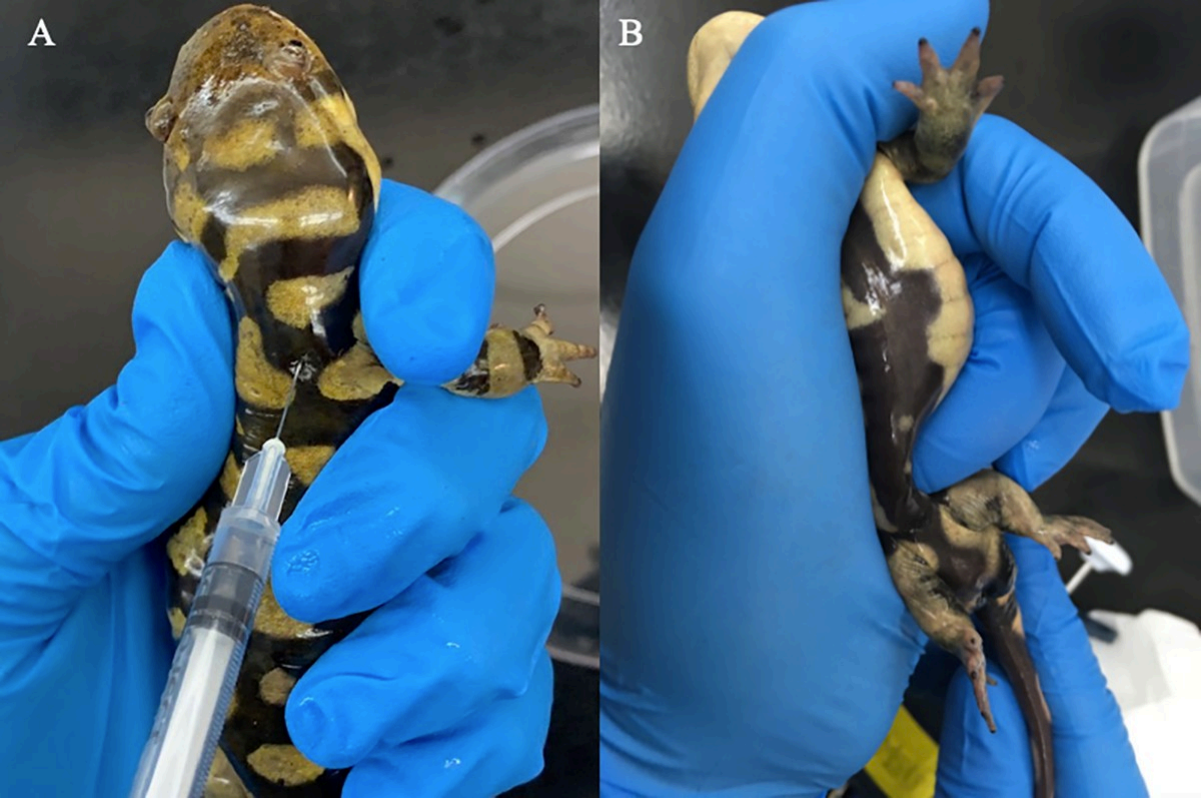
Hormone treatment and semen collection. Exogenous hormone treatment (A) by intramuscular injection and sperm collection, and (B) sperm collection through pelvic stimulation and pressure.

Salamanders were treated with varying doses of luteinizing hormone-releasing hormone analog (LHRHa; Product #L4513; Sigma Aldrich, St. Louis MO, USA), based on age and season, as previously described by Marcec [8]. Dosages of LHRHa varied between animals (largely dependent on age) and were chosen based on results from previous pilot studies conducted with these specific salamanders [8]. In brief, animals less than two years old were administered a single dose of 0.5 μg/g body weight (BW) LHRHa outside of elevated spermatogenesis (September–December), while older animals were given two doses, 0.025 μg/g BW and 0.1 μg/g BW LHRHa, 24 hours apart. During high spermatogenesis, all dosages were halved. LHRHa was suspended in 50 μL of phosphate buffered saline (0.02M, pH 7.4, 20°C) and then administered intramuscularly at the shoulder blade (Fig 2A). Animals were then placed in individual plastic containers with 1 cm of water covering the bottom to encourage milt or urine production and the enclosure covered with a solid, opaque cover to reduce stress and visibility.

A split-plot design was completed over time with an n = 10 per treatment. The first subplot factor was collection type, milt or spermic urine, as determined at time of collection and the second subplot factor was storage temperature, 0°C (ice bath) or 20°C (room temperature), applied immediately after collection. The block was defined as individual animal and the experimental unit as each sperm sample. Sperm sample collection was attempted at various times over 72 hours until the appropriate sample type was obtained with an initial total motility of > 40%. Once a sperm sample of the appropriate type was collected, the initial motility at time of collection (Time 0) was recorded, the sample split into two aliquots, and stored at either 0°C or 20°C. Subsamples of collection type, stored under both temperature treatments, were removed at designated times (0.5, 1, 2, 3, 5, 7, and 24 hours) and reevaluated for motility decay, as described below. These time points were chosen to best cover the periods when sperm samples would be cryopreserved or utilized for in-vitro fertilization. Milt was defined as a viscous, opaque substance with a high sperm concentration requiring a dilution of > 1:100 for analysis (Fig 1B), while spermic urine was described as a free-flowing, translucent solution with a lower sperm concentration, thus needing a dilution of < 1:100 (Fig 1C). Food was withheld until two days post-sperm collection.

### Sperm collection and evaluation

Sperm collection began 0.5 hours after hormone treatment and continued until an adequate sample type (milt or spermic urine) was obtained. Animals were checked again at 1 hour, every subsequent two hours for the first day, and every four hours on the following two to three days. Collections were attempted until each individual produced both a spermic urine and milt sample type of motility > 40%. Sperm collection techniques are similar to those previously described in Mansour [9] and Marcec [8] (Fig 2B). Animals were held ventral side up and blotted dry, followed by rubbing of the cloaca and massaging of the dorsal sides for approximately ten seconds each. Pressure was then applied at the pelvis (Fig 2B) and milt or spermic urine was collected directly from the cloaca with a micropipette (Fig 1B and 1C) and transferred into an Eppendorf tube. Motility was immediately evaluated upon collection. If sperm samples were too concentrated (> 1 x 10^6^ sperm/mL) for analysis, a subsample was diluted in extender, 2% trehalose + 0.2% bovine serum albumin (BSA), to maintain an isotonic environment for motility evaluation. The sample was mixed by pipetting up and down and then a 5 μL aliquot was placed on a slide without a coverslip and assessed under phase-contrast microscopy (Olympus CX31) with a 20X objective. Measurements were conducted by two researchers in an alternating manner to account for potential bias and were performed by randomly counting 100 sperm cells. Sperm cells moving with velocity in a circular motion were categorized as “progressive motile” (PM), those demonstrating an undulating membrane on the tail but not moving in a circular motion were counted as “non-progressive motile” (NPM). Cells without any of the above two signs of movement were labeled as “non-motile,” as described by Marcec [8]. Following the initial motility measurement, sperm samples were divided into two parts, with one half placed in an ice bath (0°C) and the other placed on the benchtop at room temperature (20°C). A subsample from each was then reassessed for motility, as above, at 0.5, 1, 2, 3, 5, 7, and 24 hours post-collection. Total motility (TM) was calculated for each sample as the sum of progressive motile and non-progressive motile percentage values.

### Data analysis

Assumptions of normality and homogeneity of variance were checked using the Shapiro-Wilk test and visual inspection of residual plots, respectively. Percentage data were transformed using an arcsin-square-root transformation before analysis. Changes in PM, NPM, and TM throughout the time course were analyzed using a linear mixed model repeated-measures ANOVA through the PROC MIXED procedure. The model statement included individual, sample type (milt or spermic urine), time, and temperature. For TM, the interaction of type by time was also included. Other interactions were not significant (*p* > 0.05) and therefore removed. The Time 0 collection point was not included in the dataset because temperature treatments had not yet been applied. An unstructured covariance structure was used based on the selection of the best-fit model. In this case, the unstructured covariance is most likely the best fit due to the evaluation time points not being uniformly distributed and, therefore, variances changing within subject by time point. The `unstructured' structure specifies no patterns in the covariance matrix and is completely general. Differences between the means were considered significant at *p* < 0.05 and all data are presented as mean ± SEM (Proportional SEM = sqrt(p*(1-p)/n)), unless otherwise noted. Statistical analysis was conducted using SAS version 9.4 (SAS Institute Inc., Cary, NC, USA).

## Results

### Progressive motility

**Fig 3:**
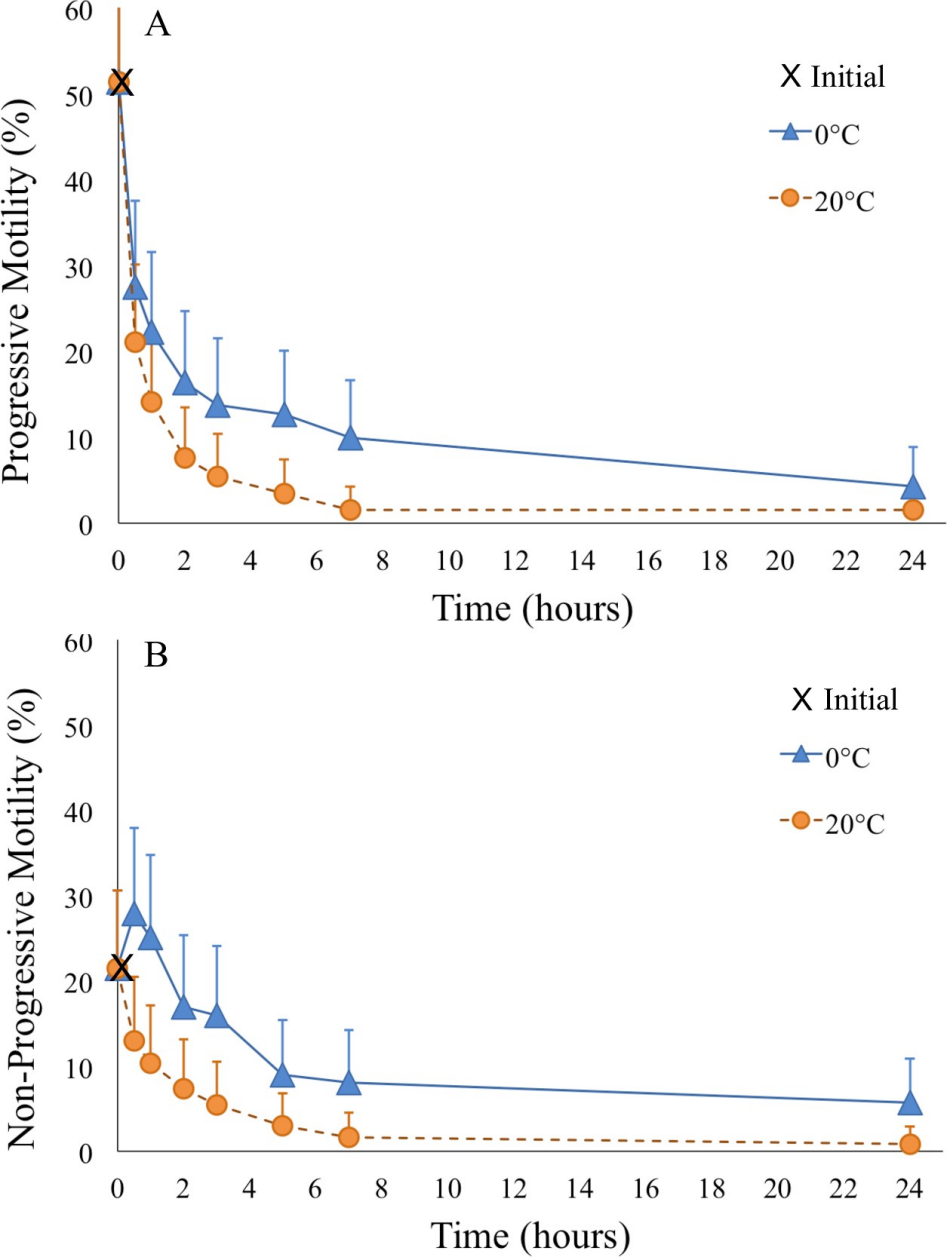
Progressive and non-progressive motility. Progressive motility (A) and non-progressive motility (B) over time of pooled milt and spermic urine samples when stored at 0°C and 20°C.

There was no effect of collection type on PM (df = 1, F-value = 2.60, *p* = 0.1015) so values were pooled within temperature at each time point (Fig 3A, S1 Data). Sperm PM was significantly (df = 6, F-value = 22.30, *p* < 0.0001) affected by time. Mean motility exhibited a rapid decrease within 30 min from 51.4 ± 11.2% initially, to 27.6 ± 10.0% when held at 0°C compared to 21.1 ± 9.1% when held at 20°C. The decay in PM of sperm held at 0°C leveled off between 2–5 hours to an average of 17.8–18.7%, and between 2–3 hours for sperm held at 20°C averaging 7.1–10.5%, after which PM began to decline again in both cases (Fig 3A). After 24 hours, only 4.3 ± 6.4% and 1.5 ± 3.9% progressive motile sperm remained when stored at 0°C and 20°C, respectfully. There was a significant effect of temperature on sperm PM (df = 1, F-value = 17.10, *p* = 0.0003) with cold temperature preserving PM in approximately 3–9% more sperm compared to room temperature at all time points after 0.5 hours post-collection. Only main effects were significant.

### Non-progressive motility

Similar to PM, collection type did not affect NPM (df = 1, F-value = 0.74, *p* = 0.3941) so values were pooled within temperature at each time point (Fig 3B). There was a significant effect of time on NPM (df = 6, F-value = 16.52, *p* < 0.0001) from the initial 21.5 ± 12.9%, but this parameter exhibited a lower rate of decay than observed for PM. Sperm held at 20°C did not show a decrease in NPM until 1 hour post-collection (10.3 ± 9.6%) while sperm held at 0°C maintained their NPM for 2–5 hours (9.0 ± 9.0%), before NPM began to decline again (Fig 3B). After 24 hours, only 5.7 ± 7.3% and 0.85 ± 2.9% of sperm exhibited NPM when stored at 0°C and 20°C, respectfully. Thus, temperature affected NPM (df = 1, F-value = 25.53, *p* < 0.0001) such that approximately 5–15% more sperm were motile under cold storage than when stored at room temperature. Again, only main effects were significant.

### Total motility

**Fig 4:**
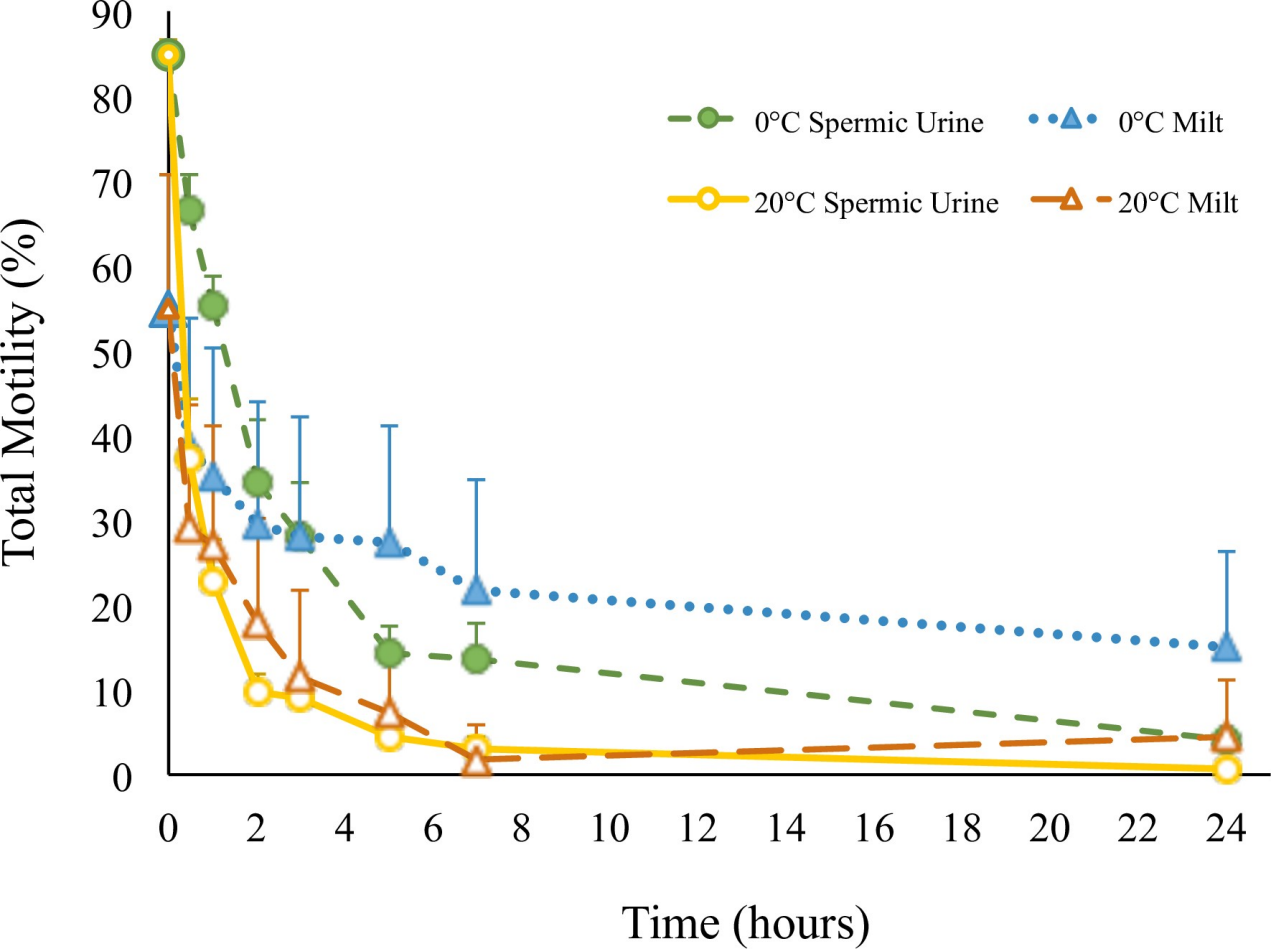
Total motility. Total motility over time of milt and spermic urine when each collection type is stored at 0°C and 20°C.

Total motility was significantly different depending on the collection type and across time (df = 6, F-value = 3.51, *p* = 0.0050, Fig 4). In spermic urine, TM (84.9 ± 11.5%) was initially higher than in milt (55.0 ± 15.7%) but declined more rapidly with time. At 0°C, the total sperm motility of either sample type decreased by approximately 20% within the first half hour after collection. Milt samples maintained 30–40% sperm motility over the next 5 hours and decreased to 14.9% after 24 hours (Fig 4). In contrast, spermic urine samples continued to decline in TM with time, dropping below the observed sperm motility of milt samples at 5 hours and eventually declining to an average TM < 5%. By comparison, at 20°C, milt sperm TM declined by 25.8% and spermic urine fell by 47.5% within the first 0.5 hour post-collection. Spermic urine TM dropped below that of milt after only 2 hours when sperm were held at 20°C. At 24 hours, TM of milt at 0°C (14.9 ± 11.2%) remained significantly higher than the other treatments (0.7–4.6%), which did not differ from each other. Other interactions were not significant (*p* > 0.05). Temperature affected TM (df = 1, F-value = 26.91, *p* < 0.0001) with cold temperature resulting in higher motility compared to room temperature, by 8.0–19.9% for milt and 3.4–32.4% for spermic urine.

## Discussion

This is the first study to describe the differences in sperm motility between milt and spermic urine in an internally fertilizing caudate, the tiger salamander. Moreover, we show there is a relationship between temperature and motility over time, regardless of sample type collected. For all types of motility (i.e., PM, NPM, and TM), longevity is extended by cold storage at 0°C compared to storage at 20°C. These results clearly show that for both milt and spermic urine, total motility decreases rapidly within the first half hour post collection, indicating samples should be used as soon as possible to maximize motility for IVF or cryopreservation.

Total motility remained above 10% up to 24 hours for milt at 0°C. Our findings are consistent with results from Marcec [8], where tiger salamander milt stored at 4°C maintained motility around 10% until 24 hours. In addition, that study continued to measure motility through 48 hours when it decreased to nearly 0%, which presumably would have occurred here as well. Marcec [8] also showed a 20% drop in total motility within the first half hour, similar to what has been observed here for both milt and spermic urine samples. Additionally, Mansour et al. [9] reported that *Ambystoma mexicanum* sperm motility, of unspecified collection type, was sustained for up to 4 hours when sperm was held at 4°C, but only for 1 hour at room temperature, which is similar to the results for spermic urine in this study.

In other caudate families such as Cryptobranchidae and Salamandridae, cold storage also led to prolonged motility, but for even longer durations than what we observed [10–12, 17]. In *Pleurodeles waltl*, sperm stored at 4°C had a high motility for over 24 hours and remained motile for nearly 120 hours [11]. In comparison, *Cryptobranchus alleganiensis* [17] and *Andrias davidianus* [10, 12] sperm held at 0–5°C was reported to maintain high motility up to 3 or more days; whereas when held at room temperature motility was reduced. Thus, our findings for Ambystomatidae are similar to what has been previously reported for two other families within the order Caudata.

In anurans, low temperatures decreased the velocity of sperm but increased the length of time sperm remained motile, potentially signifying a decrease in enzymatic activity required for flagellar motion of the tail [22]. Browne et al. [13] has suggested that low temperature induces a slowing of metabolism such that energy demanding mechanisms decline along with the rate of depletion of energy substrates, such as adenosine triphosphate (ATP), allowing for extended motility. However, findings in several fish species demonstrate that sperm velocity, motility, and enzyme activity may respond optimally at cold temperatures, especially in species that naturally spawn at 2–8°C. For *Salmo trutta*, *Lota lota* and *Thymallus thymallus*, lower temperatures promote higher sperm velocities and overall motility, as enzyme activity is maximized at the natural temperature range of the species [23]. The trends we observed here in quickly decreasing PM and increased longevity indicate that caudate sperm behaves similar to that of anuran amphibians rather than cold-spawning fish in response to temperatures near 0°C. Additionally, cold solutions hold higher concentrations of dissolved oxygen, which contributes to the continuation of ATP synthesis, reducing the depletion rate of ATP in fish sperm during storage [24, 25]. Silla et al. [26] previously found oxygen supplementation to be beneficial to sperm longevity in the frog *Litoria booroolongensis*. Likewise, Germano et al. [16] showed that aeration for 24 hours at 4°C, presumably inducing oxygen saturation of the storage medium, increased the time sperm remained motile in *Anaxyrus fowleri*.

In this study, an interaction was found between collection type (milt or spermic urine) and time affecting total sperm motility; inhibition/activation factors, diluent toxicity, and metabolism may explain this interaction. Sperm inside of a spermatophore have zero to moderate activity [8, 27], potentially due to unknown inhibition factors. It may be that milt has a higher concentration of spermatophore biochemical components than spermic urine, which might inhibit motility initially yet prolongs its survival by maintaining a low state of metabolism within the sperm cells. Cryptobranchid species naturally secrete a milt-like substance, which may account for the greater longevity of their sperm as well [10, 12, 17]. Additionally, trehalose has now been found to be toxic to *A*. *tigrinum* sperm motility at higher concentrations (10%; Gillis unpublished data) and could have caused the lower initial motility of the milt since spermic urine requires less dilution for analysis. However, other diluents tested supported similar sperm motilities when compared to trehalose, with the exception of urine, where sperm motility was nearly double.

Higher initial sperm motility in urine may be due to a higher concentration of ions that are relevant to early motility in caudate sperm, such as sodium, potassium, and calcium, also found in egg-jelly [27, 28]. A drop in osmolality does not account for sperm activation in caudates, as it does in fish and anurans [8, 28], possibly due to most caudates having internal fertilization. The optimum osmolality for caudate milt and spermic urine appear to be very similar to anurans and are within the range of 20–80 mOsmol/kg, although motility continues to be present even at physiological levels of 300 mOsmol/kg. The elevated initial sperm motility in urine may imply a higher metabolic rate exhausting stored energy or available oxygen. The advanced loss in motility, membrane damage and resulting cell death might increase reactive oxygen species (ROS) production leading to a compounding effect on loss of motility compared to milt. Moreover, sperm quickly settle to the bottom of Eppendorf storage tubes when collected as spermic urine due to the low viscosity, large urine volume, and slow forward movement of the cells, potentially creating an anoxic environment with a higher concentration of ROS, thereby reducing motility faster. In contrast, sperm stay suspended in milt much longer, possibly improving oxygenation and reducing toxicity due to lower concentration of ROS.

Bacterial contamination may also contribute to the interaction of collection type and time on total motility. For instance, bacterial loads may be higher in spermic urine because it usually occurs in larger volumes and at a higher rate of excretion than milt, potentially clearing more bacteria from the cloacal colonies. Bacteria present in the cloaca and excreted into the urine can negatively impact sperm motility, as observed in fish [29, 30], by competing for resources within the storage medium and producing enzymes that can cause cell rupture and death. While antibiotics have either had no effect or been a detriment to anuran sperm [16, 26, 31], neither bacterial growth nor antibiotic use have been studied in caudate spermatozoa. We did not directly measure bacterial growth in our stored samples; however, damage to the tail membrane of spermatozoa could be seen by 24 hours in spermic urine stored at room temperature. Damage to sperm membranes was not evident in samples stored at 0°C for 24 hours, possibly due to slower bacterial growth at cooler temperatures. Further investigation is needed to understand the cause of injury to the sperm tail membranes when stored at the two different temperatures.

In conclusion, this study demonstrates important differences in motility between Ambystomid milt and spermic urine over the first 24 hours post-collection and the positive effect of cold storage on each. While the collection of milt or spermic urine was not controllable in our study, our results suggest that both collection types should be stored at 0°C and the sperm used as early as possible for IVF and cryopreservation. Additionally, milt should be prioritized past three hours post-collection when both sample types are available due to its higher sustained total motility, especially for overnight transportation between institutions. While species differences may exist in overall longevity and optimum temperature, the recommendation for sperm cold storage holds across all amphibia studied to date. Application of cold storage to future salamander ART could enhance fertility and genetic management of endangered, captive and wild populations. Yet, future research is required to address implications to caudate sperm metabolism, activation, and bacterial contamination.

## Supporting information

S1 Data Sperm motility data. Progressive, non-progressive, and total motility data for both collection types at all time points.
(XLSX)
